# Analysis of Phenotypic Characteristics and Sucrose Metabolism in the Roots of *Raphanus sativus* L.

**DOI:** 10.3389/fpls.2021.716782

**Published:** 2021-10-21

**Authors:** Ji-Nam Kang, Jung Sun Kim, Si Myung Lee, So Youn Won, Mi-Suk Seo, Soo-Jin Kwon

**Affiliations:** Genomics Division, National Institute of Agricultural Sciences, Rural Development Administration, Jeonju, South Korea

**Keywords:** sucrose metabolism, sugar content, phenotypic analysis, sucrose synthases, radish roots

## Abstract

The taproot of radish (*Raphanus sativus* L.) is an important sink organ; it is morphologically diverse and contains large amounts of secondary metabolites. Sucrose metabolism is believed to be important in the development of sink organs. We measured the amounts of glucose, fructose, and sucrose in the roots of sixty three radish accessions and analyzed the association between the sugar content and the root phenotype. Fructose content correlated with the root color and length characteristics, glucose was the most abundant sugar in the roots, and the sucrose content was very low, compared to that of the hexoses in most of the accessions. Expression analysis of the genes involved in sucrose metabolism, transportation, starch synthesis, and cell wall synthesis was performed through RNA sequencing. The genes encoding sucrose synthases (SUSY) and the enzymes involved in the synthesis of cellulose were highly expressed, indicating that SUSY is involved in cell wall synthesis in radish roots. The positive correlation coefficient (*R*) between the sucrose content and the expression of cell wall invertase and sugar transporter proteins suggest that hexose accumulation could occur through the apoplastic pathway in radish roots. A positive *R* score was also obtained when comparing the expression of genes encoding SUSY and fructokinase (FK), suggesting that the fructose produced by SUSY is mostly phosphorylated by FK. In addition, we concluded that sucrose was the most metabolized sugar in radish roots.

## Introduction

Radish (*Raphanus sativus* L.) is a nutritionally important root crop belonging to the Brassicaceae family, which includes cabbage, kale, and broccoli. The roots of radish are mainly used for human consumption in the East Asian countries, including Korea and Japan (Kang et al., 2020). In particular, the taproot of radish contains large amounts of secondary metabolites, minerals, vitamins, and carbohydrates, which is its most remarkable trait (Mitsui et al., 2015). The phenotypes of radish roots are well characterized; radish roots are diverse in shape, longitudinal section, length, diameter, weight, skin surface texture, and skin color (International Board for Plant Genetic Resources, and Commission of the European Communities, 1990; Mitsui et al., 2015).

**FIGURE 1:**
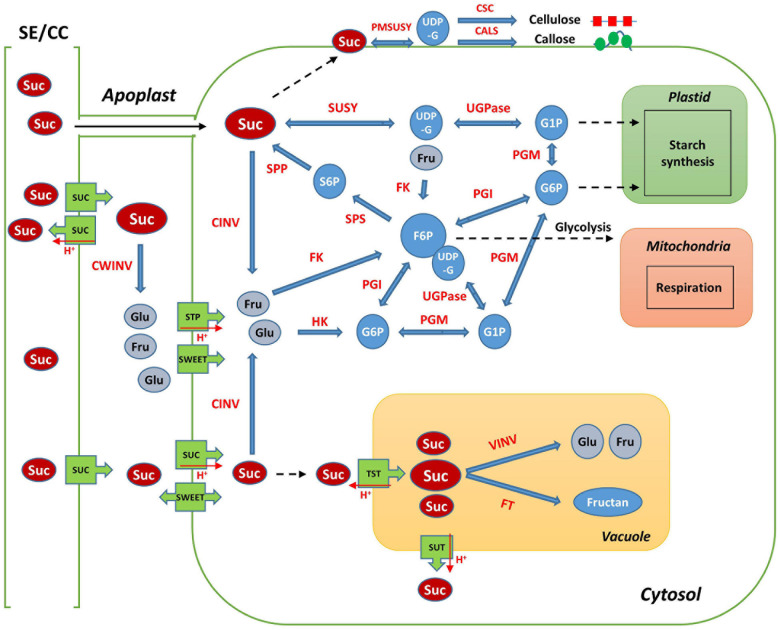
Schematic map of sucrose metabolism in plant sink cell. SE/CC, sieve element/companion cell; Suc, sucrose; Glu, Glucose; Fru, Fructose; UDP-G, uridine diphosphate glucose; G1P, glucose-1-phosphate; G6P, glucose-6-phosphate; F6P, fructose-6-phosphate; S6P, sucrose-6-phosphate; SUSY, sucrose synthase; CWINV, cell-wall invertase; CINV, cytosol invertase; VINV, vacuole invertase; UGPase, UDP-glucose pyrophosphorylase; PGM, phosphoglucomutase; PGI, phosphoglucose isomerase; FK, fructokinase; HK, hexokinase; SPS, sucrose-6-phosphate synthase; SPP, sucrose-phosphate phosphatase; VINV, vacuolar invertase; FT, fructosyltransferase; SUC, sucrose transporter protein; SWEET, sugars will eventually be exported transporter; STP, sugar transporter protein; TST, tonoplast sucrose transporter; PMSUSY, plasma membrane-associated SUSY; CSC, cellulose synthase complex; CALS, callose synthase.

Sucrose, a disaccharide, is either broken down into monosaccharides for metabolism in the sink organ or stored in the form of starch and cellulose, in most of the higher plants. Sucrose is also stored specifically in the stems or roots, such as in sugar cane and sugar beets (McCormick et al., 2009; Stein and Granot, 2018). Sucrose is the final product of photosynthesis and is the main form of carbon generally transported from the “source” to the “sink” organ through the phloem, in higher plants (Yu et al., 2016; Stein and Granot, 2019). Sucrose metabolism is considered essential for root growth and development in radish, because it is active when the tuberous roots begin to develop. In addition, it is a signal molecule regulating the expression of transcription factors, microRNAs, plant hormones, and many other genes (Stokes et al., 2013; Xiong et al., 2013; Mitsui et al., 2015; Yu et al., 2016). The main pathways and enzymes involved in sucrose metabolism in plants are well described (Figure 1; Wang et al., 2016; Verbančič et al., 2018; Stein and Granot, 2019). When sucrose arrives at sink tissues, it is transported from the sieve element/companion cell complex (SE/CC) to the apoplast; it is then hydrolyzed by cell wall invertase (CWINV) to produce glucose and fructose. Sucrose can also pass directly from the phloem to the cytosol through plasmodesmata (Stein and Granot, 2019). In the cytosol, sucrose is cleaved by sucrose synthase (SUSY) to generate uridine diphosphate glucose (UDP-G) and fructose, while cytosolic invertase (CINV) can hydrolyze sucrose to glucose and fructose, which are then converted to glucose-6-phosphate (G6P) and fructose-6-phosphate (F6P) by hexokinase (HK) and fructokinase (FK), respectively (Stein and Granot, 2019). UDP-G is converted to glucose-1-phosphate (G1P) by UGPase. G1P and G6P are transferred into plastids and are used mainly for starch biosynthesis (Wang et al., 2016). Phosphoglucose isomerase (PGI) and phosphoglucomutase (PGM) are involved in the transformation of G6P to F6P, which can convert G1P to UDP-G. F6P is used to resynthesize sucrose by combining it with UDP-G in the cytosol. This complex is converted to sucrose-6-phosphate (S6P) by sucrose-6-phosphate synthase (SPS), S6P can be dephosphorylated by sucrose-phosphate phosphatase (SPP) to form sucrose (Stein and Granot, 2019). G6P and F6P can also be used for glycolytic respiration in the cytosol and plastids (Granot et al., 2013; Stein and Granot, 2018).

Sugar transport is a process that involves cell-to-cell transport and transport between multiple organelles such as the phloem, apoplast, cytosol, and vacuole. The accumulation, compartmentalization, and storage of sugar in plants is regulated by sugar transporters such as sucrose transporter protein (SUC), monosaccharide transporter (MST), and SWEET (Sugars Will Eventually be Exported Transporter) (Eom et al., 2015; Wang et al., 2016; Julius et al., 2017). SUCs are H^+^-coupled symporters and are essential for the translocation of sucrose via phloem loading (Chen, 2014; Julius et al., 2017). The MST family includes the sugar transporter protein (STP) and tonoplast monosaccharide transporter (TMT) (Wang et al., 2016). STPs play a role in the transport of hexose from the apoplast to the cytosol (Büttner, 2007) and TMTs are responsible for sugar transport in the tonoplast (Eom et al., 2015; Jung et al., 2015). SWEETs are structurally different from other transporters such as MSTs and SUCs; they are responsible for the secretion of sucrose as a prerequisite for SUC1-mediated phloem loading (Chen, 2014; Eom et al., 2015).

Sucrose is not only the source of carbon skeletons, but is also important in the biosynthesis of essential metabolites, such as starch and cellulose (Yu et al., 2016). SUSY is a potential factor responsible for cell wall production, because it directly supplies UDP-G to cellulose and callose synthases (Amor et al., 1995; Persia et al., 2008; Verbančič et al., 2018). CINV also contributes to cellulose synthesis by supplying UDP-G (Rende et al., 2017; Barnes and Anderson, 2018). Starch synthesis is occurred through the catalysis of SUSY and UDPase in sink organs (Kleczkowski et al., 2004; Zhang et al., 2017; Stein and Granot, 2019). Cellulose and starch are the most abundant biosynthetic compounds in the world and are the primary carbon sinks in plants (Baroja-Fernández et al., 2012). Starch synthesis commonly occurs in amyloplasts, catalyzed by starch synthase (SS), whereas cellulose and callose are synthesized in the plasma membrane by cellulose synthase (CESA) and callose synthase (CALS), respectively, for cell wall formation in sink organs (Persia et al., 2008; Noronha et al., 2018; Stein and Granot, 2019).

Large amounts of transcriptomic information are generated for understanding the growth and development mechanisms of plants (Yu et al., 2016). Recently, the draft genome of *R. sativus* var. *hortensis* was sequenced and published (Mitsui et al., 2015). Global transcriptome analysis was performed in radish roots, using RNA sequencing (RNA-seq) (Mitsui et al., 2015; Yu et al., 2016). In this study, we focused on the association between the sugar content and the phenotypic characteristics of radish roots. In addition, we tried to understand the sugar accumulation by analyzing the expression of genes involved in sucrose metabolism. The amount of the individual sugars in the roots of sixty three radish accessions was investigated, and the expression of genes involved in sucrose metabolism, sugar transport, starch synthesis, and cell wall synthesis were comprehensively analyzed using RNA-seq.

## Materials and Methods

### Radish Materials and Sample Preparation

The radish accessions used in this study were the same as that from our previous study (Kang et al., 2020). Seeds of sixty three radish accessions were collected from the National Agrodiversity Center (Korea, IT-), the Leibniz Institute of Plant Genetics and Crop Plant Research (Germany, RA-), and the National Agriculture and Food Research Organization (Japan, JP-). Seven young plants from each accession were grown in plastic pots and transferred to the field for plant growth. After 8 weeks, three independent plants from each accession were gently harvested from the ground, and the morphological characteristics of the taproot, including length, weight, and color, were recorded. The central part of the taproot was cut into two pieces. One piece was freeze-dried for sugar content analysis and the other piece was stored at −80°C for RNA sequencing.

### Determination of Individual Sugar Content

High-performance anion-exchange chromatography (HPAEC) was used for quantifying the reducing sugars. A mixture of 100 mg freeze-dried powder of radish roots and 1.5 mL of 5% trichloroacetic acid (TCA) solution was stirred thoroughly for 1 min. The mixture was centrifuged (5 min at 13,000 × g), the supernatant was filtered, and the eluent was used for the quantitative analysis of reducing sugars. The soluble sugar content was analyzed using a PHAEC system (Dionex, United States) with a CarboPac^TM^ PA1 column (4 mm × 250 mm). Mobile phases A and B contained 150 mM NaOH and 600 mM sodium acetate with 150 mM NaOH, respectively. The flow rate was 1 mL/min and separation was performed under the following gradients: 0–5 min, 0% B; 5–10 min, 10% B; 15–20 min, 100% B; 20–30 min, 0%. The concentration of individual soluble sugars was quantified against the standards for sucrose, glucose, and fructose.

### RNA Sequencing and Gene Annotation

Total RNA was isolated from the roots of two radish accessions, JP-61 and JP-64, using a Hybrid-R kit (GeneAll, Korea), according to the manufacturer’s instructions. The quality of the RNA was assessed; 2 μg of total RNA was used to construct RNA-seq libraries and libraries with an insert size of 300 bp were generated using an Illumina TruSeq RNA Sample Preparation Kit (Illumina, United States), according to the manufacturer’s instructions. The Illumina HiSeq X platform was employed for RNA-seq at Macrogen Co. (Seoul, Korea), and paired-end reads of 101 bp were obtained from the pooled libraries. Mapping of reads and gene annotation was performed according to Kang et al. (2020). The adapter sequences followed by the low quality and duplicated reads were removed using the Trimmomatic program (ver. 0.38). High-quality RNA-seq reads were mapped using HISAT2^1^ in the coding region of the radish genome sequences of *R. sativus* var. *hortensis* cv. Aokubi is a double haploid line (Mitsui et al., 2015). The mapped reads were counted using HTSeq-count^2^ and the fragments per kilobase of transcript per million (FPKM) values were calculated for the gene expression analysis. Gene annotation was performed using nrBLAST, InterProScan, and Araport11 for predicting the gene function. Sequencing data for the IT-15, RA-82, RA-92, RA-74, and IT-8 accessions, excluding JP-61 and JP-64, were obtained from our previous study (Kang et al., 2020). Information on genes involved in sucrose metabolism, transportation of sugar, synthesis of cellulose and callose, and starch synthesis was obtained from previous studies (Jung et al., 2015; Wang et al., 2016; Yu et al., 2016; Julius et al., 2017; Stein and Granot, 2019).

## Results

### Analysis of Sugar Content and Phenotype Characteristics in Radish Roots

Sixty three radish accessions, including *R*. *sativus* var. *sativus*, *R*. *sativus* convar. *sativus* (radish group), *R*. *sativus* convar. *sativus* (small radish group), *R*. *sativus* var. *longipinnatus* (daikon group), *R*. *sativus* convar. *caudatus*, and *Raphanus* sp. were used for the analysis of sugar content and the phenotypic characteristics of radish roots (Supplementary Figure 1). Fructose, glucose, and sucrose were detected in the roots. The median value of fructose was 90.32 mg⋅g^–1^ dry weight (DW) within the range of 7.02–310.91 mg⋅g^–1^ DW, and that of glucose was 137.65 mg⋅g^–1^ DW within the range of 25.07–292.89 mg⋅g^–1^ DW. The median value of sucrose was 25.18 mg⋅g^–1^ DW in the range of 0–200.02 mg⋅g^–1^ DW. Glucose, fructose, and sucrose accounted for 47.15, 30.87, and 8.58% of the total content, respectively, based on the median values (Supplementary Figure 2A). The total DW and root length were distributed in the range of 1.11–61.39 g and 4.33–37.60 cm and their median values were 22.39 g and 18.93 cm, respectively. The diameter of central part of the taproot was in the range of 1.97–12.9 cm, and the median value was 6.3 cm (Supplementary Figure 2B). Skin color types, including white, pink, brown, and green, were identified among the sixty three radish accessions (Supplementary Figure 1). Detailed information on sugar content and phenotypic characteristics is indicated in Supplementary Table 1.

### Correlation Analysis Between the Sugar Content and Root Phenotypes

**FIGURE 2:**
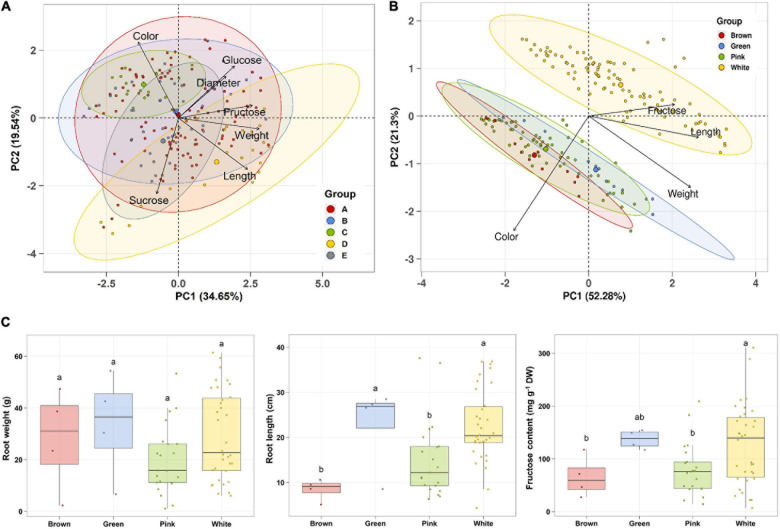
Association analysis between sugar content and root morphological characteristics. Data visualization performed using R program. **(A)** PCA plot based on five radish groups. A, *Raphanus sativus* var. *sativus*; B, *Raphanus sativus* convar. *sativus* (Radish group); C, *Raphanus sativus* convar. *sativus* (Small radish group); D, *Raphanus sativus* var. *longipinnatus* L. H. Bailey (Daikon Group); E, *Raphanus* sp. with *Raphanus sativus* convar. *caudatus* (L.f.) Pistrick. **(B)** PCA plot based on root color. **(C)** Box plot analysis. Anova and Tukey Honest Significance Difference (HSD) test was used to evaluate the differences between the groups. Other letters in the plot indicate significant differences at the *p* < 0.05 level.

**FIGURE 3:**
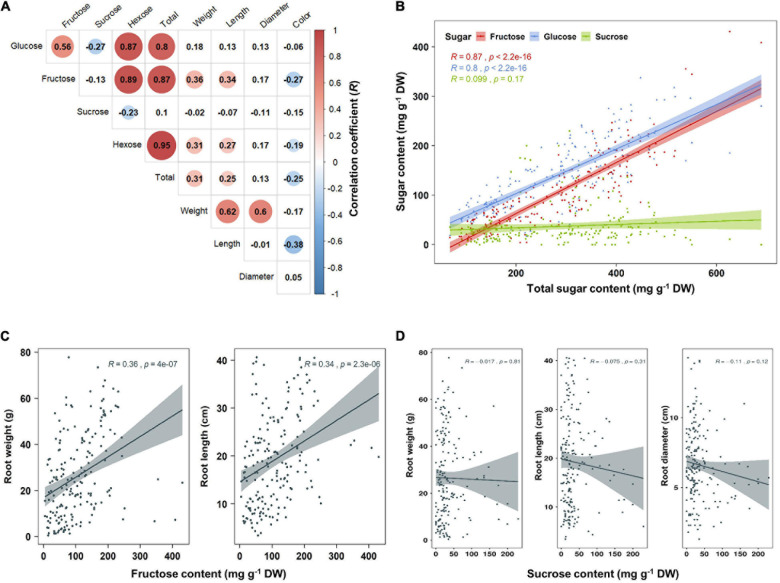
Analysis of Pearson’s correlation coefficient (*R*) between sugar content and root phenotypes. Data visualization performed using R program. **(A)** Heat map of *R* score. The *R* scores with *p* < 0.01 are indicated by color. Red and blue circles represent positive and negative *R* scores, respectively. **(B)** Correlation between total sugar content and individual sugar content. **(C)** Correlation between fructose content and root phenotypes. **(D)** Correlation between sucrose content and root phenotypes.

**FIGURE 4:**
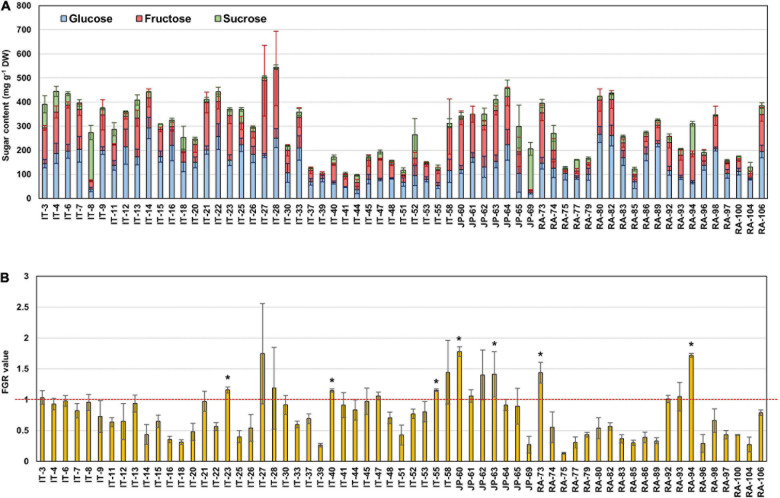
The sugar ratio in each radish root. **(A)** Distribution of glucose, fructose, and sucrose in each radish root. **(B)** Fructose to glucose ratio (FGR). The asterisks indicate that the FGR value statistically exceeds 1 by *t*-test (*p* < 0.05).

Principal component analysis (PCA) was performed using 189 radish samples (three individual samples from each accession) to confirm the association between the sugar content and root phenotypes. PC1 and PC2 represented 55.19% of the seven variables (Figure 2A). When the five radish groups were reflected in seven variables, only the small radish (C) and daikon (D) groups were distinguishable. The root phenotypes of the small radish group were spheric or transverse elliptic with pink and brown skin color, except for one accession. The daikon group showed mostly long and white root phenotypes (Supplementary Figure 1). The main variables used to separate the two groups were the length and color of the roots. The other radish groups were not divided into seven variables. Sucrose was negatively loaded into PC1 and PC2 as a single variable (Figure 2A). Similar results were identified when analyzing the sixty three radish samples, based on the mean values (Supplementary Figure 2C). Root length was positively correlated with the weight and fructose content of roots in PC1 (Figure 2A). Therefore, we changed the grouping criteria from radish varieties to root color phenotype and tried PCA using four variables, color, weight, length, and fructose content (Supplementary Figure 3 and Figure 2B). PC1 and PC2 accounted for 73.58% of the variables. Interestingly, white radish and colored radish were clearly distinguishable. Root length and fructose content were identified as variables associated with white radishes (Figure 2B and Supplementary Figure 2D). Box plot analysis was performed to verify the differences in the root weight, root length, and fructose content in relation to the root color. The weight of the white radish was not significantly different from that of the other colored groups. The length and fructose content of white radish were significantly different from that of the brown and pink radish. Green radish roots were similar to that of white radish in terms of weight, length, and fructose content. The roots of white radish were significantly longer and had higher fructose content, compared to that of brown and pink radish (Figure 2C). Correlation coefficient (*R*) analysis supported these results. The total sugar content showed a positive *R* score with hexose content (Figures 3A,B). The fructose content showed a positive *R* score with weight and length, and a negative *R* score with the color of roots (Figures 3A,C). Root diameter did not show a significant *R* score with sugar content (Figure 3A). Root color showed a negative *R* score with the root length. Glucose and sucrose content did not show significant *R* scores for root phenotypes (Figures 3A,D). The fructose-to-glucose ratio (FGR) was used to analyze the distribution pattern of hexose in radish roots. Sucrose was the dominant sugar only in IT-8 and JP-69 (Figure 4A). The FGR value was equal to or significantly less than one in most of the accessions. Only seven accessions statistically exceeded the FRG value of one (Figure 4B). These results indicate that glucose is the main sugar and that fructose content is associated with the phenotypic characteristics of radish roots, such as weight, length, and color.

### Expression Analysis of Genes Involved in Sucrose Metabolism in Radish Roots

**FIGURE 5:**
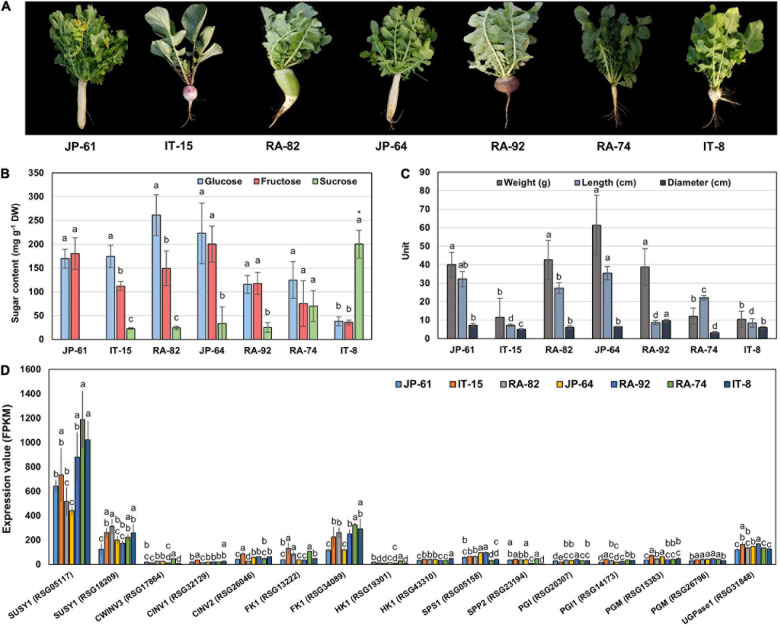
Information of phenotypes, sugar content, and genes expression involved in sucrose metabolism in seven radish roots. The error bars represent the standard deviation of three repeated data. **(A)** Phenotypic images. **(B)** Individual sugar content. Anova and Tukey HSD were performed to reveal the differences of individual sugar content in each accession. Other letters above the bar graph indicate significant differences at the *p* < 0.05 level. **(C)** Measurement of the morphological characteristics. Other letters above the bar graph indicate significant differences at the *p* < 0.05 level in phenotypic characteristics between each radish accession. **(D)** Expression results of genes involved in sucrose metabolism. Anova and Tukey HSD were used to evaluate the differences between the sucrose metabolism genes expressed in each accession. The letters above the bar graph indicate significant differences at the *p* < 0.05 level.

To identify the genes related to sucrose metabolism in radish roots, we selected seven radish accessions (Figure 5A). JP-61, IT-15, RA-82, JP-64, and RA-92 had high hexose and low sucrose content. RA-74 had similar hexose and sucrose contents. IT-8 had a significantly higher sucrose content compared to the hexose content and it had the highest sucrose content and was an extreme outlier among the sixty three radish accessions (Supplementary Figure 2A and Supplementary Table 1). The phenotypic characteristics of the seven radish roots are shown in Figure 5C. Correlation analysis between the sugar content and root phenotypic characteristics was conducted using 21 radish samples (three individual samples from seven accessions). Hexose had a positive *R* score for total sugar content, and fructose showed a positive *R* score for the weight and length of roots. Sucrose content did not show a significant *R* score with any of the phenotypic characteristics (Supplementary Figure 4).

We conducted RNA-seq analysis of the radish accessions. The sequencing and mapping results were presented in Supplementary Table 2. Eighty one genes involved in sucrose metabolism were identified in radish roots. These genes encoded for 10 main enzymes, SUSY, CWINV, CINV, FK, HK, UDPase, PGI, PGM, SPS, and SPP (Supplementary Table 3). Two SUSY1-encoding genes (*RSG05117* and *RSG18209*), one CWINV3-encoding gene (*RSG17864*), two CINV-encoding genes (*RSG32129* and *RSG26046*), two FK1-encoding genes (*RSG13222* and *RSG34089*), two HK1-encoding genes (*RSG19301* and *RSG43310*), one UGPase-encoding gene (*RSG31848*), two PGI-encoding genes (*RSG20307* and *RSG14173*), two PGM-encoding genes (*RSG15383* and *RSG26796*), one SPS1-encoding gene (*RSG05158*), and one SPP2-encoding gene (*RSG23194*) showed relatively higher expression, compared to that of their ortholog genes (Supplementary Figures 5A,B). In particular, the *RSG05117* gene encoding SUSY1 showed remarkably high expression among that of all the sucrose metabolism genes identified (Supplementary Figure 5A). These 16 genes are considered responsible for sucrose metabolism in radish roots.

### Correlation Analysis Between the Sugar Content and Sucrose Metabolism Genes in Radish Roots

**FIGURE 6:**
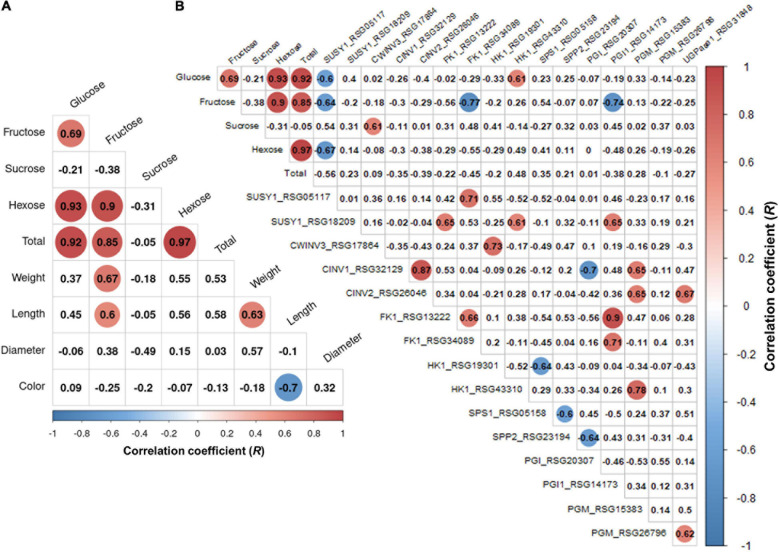
Analysis of Pearson’s correlation coefficient (*R*) among the sugar content, root phenotypes, and expression level of sucrose metabolism genes in the six radish accessions. The outlier accession IT-8 was excluded. Data visualization was performed using R program. The *R* scores with *p* < 0.01 are indicated by color. Red and blue circles represent positive and negative *R* scores, respectively. **(A)** Heat map of *R* score between sugar content and root phenotypes. **(B)** Heat map of *R* score between total sugar content and expression level of genes involved in sucrose metabolism.

**FIGURE 7:**
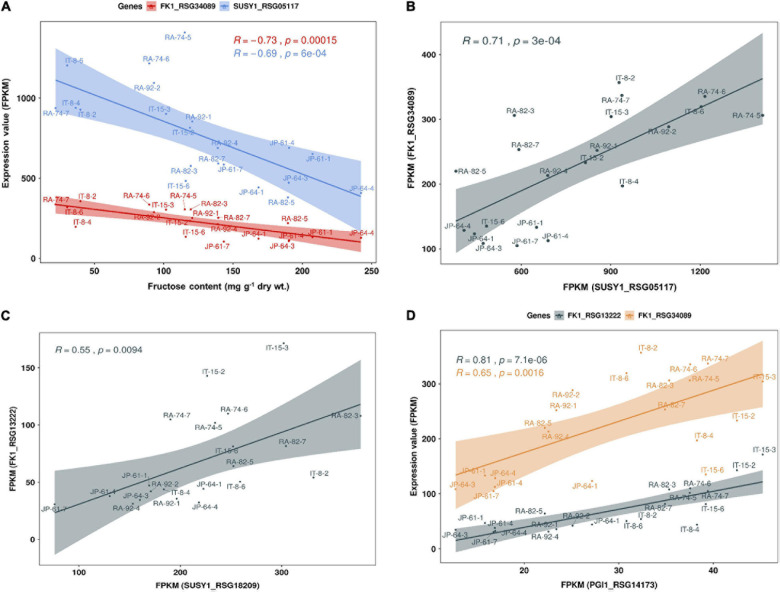
Scatter plot of Pearson’s correlation coefficient (*R*) between fructose content and expression level of genes involved in sucrose metabolism in seven radish roots. Data visualization performed using R program. **(A)** The fructose content has a negative correlation with the expression levels of SUSY1-encoding gene *RSG05117* and FK1-encoding gene *RSG34089*. **(B)** Expression level of *RSG05117* and *RSG34089* shows a positive correlation and **(C)** similar results are observed among their paralog genes, *RSG18209* and *RSG13222*. **(D)** Expression levels of the two FK1-encoding genes show a positive correlation with that of PGI1-encoding gene, *RSG14173*.

We performed a correlation analysis between the 16 major genes involved in sucrose metabolism and the sugar content in the seven radish accessions. The sucrose content of the IT-8 accession was significantly higher than that of the other six accessions; however, the expression of genes involved in sucrose metabolism was not specific for IT-8 among the tested accessions (Figures 5B,D). Expression of the SUSY1-encoding gene *RSG05117* in IT-8 was comparable to that of IT-15, RA-92, and RA-74. Another SUSY1-encoding gene *RSG18209* was similarly expressed in the tested accessions except JP-61 (Figure 5D). The sucrose content did not show any significant correlation with the expression of the sucrose metabolism genes among the tested accessions (Supplementary Figure 6). Sucrose content could be influenced by the expression of sugar transporters; and therefore, we investigated the expression levels of 85 genes encoding sugar transporters in the radish roots (Supplementary Table 4). Four SUC-encoding genes *RSG21948*, *RSG49762*, *RSG35389*, and *RSG51374*, five SWEET-encoding genes *RSG33430*, *RSG25697*, *RSG35586*, *RSG11173*, and *RSG45880*, two TMT-encoding genes *RSG15186* and *RSG04190*, and an STP1-encoding gene *RSG00099* were relatively highly expressed, compared to that of their paralog genes (Supplementary Figures 7A,B). However, sucrose content did not show a significant correlation with the expression levels of any of the sugar transporters (Supplementary Figure 7C, upper). These results indicate that the sucrose content in radish roots was not influenced by the expression levels of genes involved in sucrose metabolism, including the sucrose transporters. The sucrose content of the IT-8 was an extreme outlier; and therefore, we performed a correlation analysis after excluding IT-8 (Figure 6A). Sucrose content was significantly correlated with the CWINV3-encoding gene, *RSG17864* (Figure 6B). The fructose content showed a negative *R* score with the expression levels of the SUSY1-encoding gene *RSG05117* and the FK1-encoding gene *RSG34089* (Figure 7A). Furthermore, these genes had a positive *R* score for each other (Figure 7B). Similar results were observed for the paralog genes *RSG18209* and *RSG13222*, which encode SUSY1 and FK1, respectively (Figure 7C). In addition, the two FK1-encoding genes showed positive *R* scores with the PGI-encoding gene *RSG14173* (Figure 7D). These results suggest that the genes encoding SUSY1 and FK1 work together in radish roots and that of fructose produced by SUSY1 is sequentially metabolized by FK1 and PGI.

### Predominant Expression of Cellulose Biosynthetic Genes in Radish Roots

**FIGURE 8:**
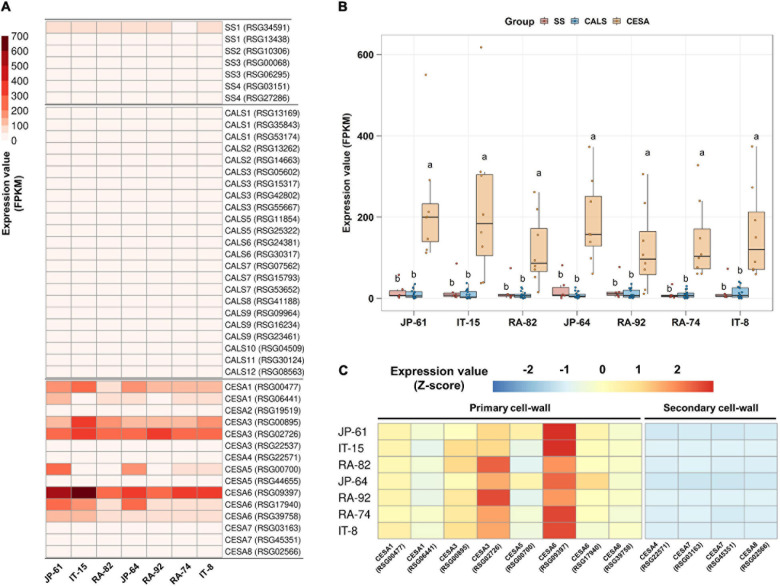
Expression analysis of starch and cell wall synthesis related-genes in the seven radish roots. Data visualization performed using R program. **(A)** Analysis of heat map using FPKM. **(B)** Box plot analysis of genes involved in the synthesis of starch, callose, and primary cell wall. Genes below 2 FPKM were excluded among the tested accessions. Anova and Tukey HSD were used to reveal the differences between the integrated expression of the genes involved in this biosynthesis in each accession. Other letters in the plot indicate significant differences at *p* < 0.05. **(C)** Relative expression analysis of genes involved in the synthesis of primary and secondary cell walls. Expression values of individual genes in each accession were calculated using the Z-score.

Two SUSY1-encoding genes were highly expressed in JP-61, where the sucrose content was undetectable (Figures 5B,D). Sugar was detected only in the soluble compartment in our study; and therefore, we hypothesized that SUSY could act in the insoluble regions such as the membrane pool. SUSY is directly associated with the synthesis of cellulose and callose in the plasma membrane and is involved in starch biosynthesis in the cytoplasm (Amor et al., 1995); and therefore, we identified seven genes involved in starch synthesis, 23 genes involved in callose synthesis, and 15 genes responsible for cellulose synthesis (Supplementary Table 5). In the tested accessions, the genes encoding CESA are highly expressed. Genes encoding CESA1, 3, 5, and 6 showed distinctly higher expression levels (Figure 8A). The integrated expression values of these genes were significantly higher than that of SS and CALS (Figure 8B). CESA1, CESA3, CESA5, and CESA6 are subunits of the cellulose synthase complex (CSC), involved in primary cell wall formation (Endler and Persson, 2011; Liepman and Cavalier, 2012; Verbančič et al., 2018). CESAs that function in secondary cell wall synthesis were expressed at low levels (Figure 8C). These results suggest that SUSY could act on the insoluble membrane, regardless of the sucrose content in the soluble regions of the cell and that it is involved in the primary cell wall synthesis of radish roots.

## Discussion

### Sugar for Plant Development

Sugar is essential for plant growth and development, and it can act as a signaling molecule and regulator of gene expression. Sucrose, glucose, fructose, and UDP-G are detected as signals in specific metabolic pathways and developmental responses in plants (Eveland and Jackson, 2012). In this study, correlation analysis showed that the color and length of radish roots were negatively correlated. Most of the pink and brown radish had roots that were shorter than that of the white radish accessions (Figure 2C). The color traits of these roots were likely caused by anthocyanin and lignin deposition. Sugars promote anthocyanin accumulation in radish hypocotyls; the supply of exogenous sucrose causes rapid and distinct anthocyanin accumulation in the radish hypocotyls compared to that of the control (Hara et al., 2003). Supply of sucrose promoted lateral root formation in anthocyanin-accumulated hypocotyls. Sucrose is a signal for anthocyanin biosynthesis and the morphological development of the hypocotyl; this is applicable to the radish hypocotyl (Hara et al., 2003). Growth and lignin synthesis showed a significant negative correlation in the hybrid forest tree population. Sucrose could be a major regulator of lignin synthesis. The negative correlation between growth and lignin deposition is caused by the competition for carbon allocation between the cellulose and lignin synthesis pathways (Novaes et al., 2010). The anthocyanin and lignin biosynthetic pathways are closely linked in plants. They are two major pathways in phenylpropanoid metabolism and share the substrate ρ-coumaroyl CoA (Hara et al., 2004; Peng et al., 2008). Distinct anthocyanin accumulation occurs under nitrogen (N) limitation, which is the ability to adapt to N limitation in Arabidopsis (Peng et al., 2008). The Arabidopsis *NLA* (nitrogen limitation adaptation) mutant did not accumulate anthocyanin in the N limitation, instead accumulating significant lignin. The authors concluded that the phenylpropanoid metabolic flux was converted from anthocyanin to lignin synthesis in the *NLA* mutant under the N limitation (Peng et al., 2008). Therefore, plant development could be affected by the sucrose signals applied to metabolic pathways other than that for plant growth. It is possible that the short morphological characteristics of the colored radish roots are partially related to anthocyanin and lignin biosynthesis. A negative correlation between root length and color of radish has also been reported in other study (Yi et al., 2016).

The fructose content showed a significant positive *R* score with root length (Figure 3A). These results can be explained based on the sink capacity of the roots. Increasing the sink capacity lowers the local concentration of sucrose in sink organs, which makes it possible to unload more sucrose from the source organ into the sink organ across a concentration gradient through the phloem (Rouhier and Usuda, 2001; Mitsui et al., 2015; Fugate et al., 2019; Stein and Granot, 2019). Therefore, the resulting fructose by sucrose degradation could accumulate in the large sink organs. Hexoses promote organ growth and proliferation in plants (Eveland and Jackson, 2012). A positive correlation between the local hexose concentration and root elongation was reported in Arabidopsis. It has been suggested that the structural properties of roots can be significantly affected by carbon availability in plant, which may be caused by local hexose concentration (Freixes et al., 2002).

### Sucrose Content in Radish Roots

The hexose content was very high, whereas the sucrose content was approximately 8.5% in the sixty three radish accessions (Supplementary Figure 2A). A distinctly high sucrose content was identified in two accessions, IT-8 and JP-69, which is possibly a transient phenomenon during the growth process. We could not identify any molecular mechanisms underlying sucrose accumulation in the IT-8 roots at the transcriptional level. In addition, the sucrose content in IT-8 and JP-69 was not high, compared to the hexose content in our previous study, using the same radish accessions. In particular, the sucrose content of IT-8 was only approximately 5.8% of the total sugar content. However, the overall sucrose content was very low, and glucose was the main sugar among the 82 radish accessions (Seo et al., 2018). These results suggest that sucrose is not the main sugar and is mostly metabolized in radish roots.

The sucrose content of the IT-8 roots was unique among the sixty three radish accessions. The initial approach of this study was to identify a specific molecular mechanism that influences the high sucrose content in IT-8 roots. Invertases, SPS, and SPP are responsible for the storage and re-synthesis of sucrose in sugar cane stalk, and SUSYs are involved in the storage of sucrose in sugar beet roots (Giaquinta, 1979; Wang et al., 2013; Fugate et al., 2019). SUC1 and SUC2 are responsible for apoplastic phloem unloading and the cellular uptake of sucrose into sink cells (Wang et al., 2016; Julius et al., 2017). Clade III SWEETs are efficient sucrose transporters (Eom et al., 2015; Julius et al., 2017). SUC4 and TMT are sucrose transporters located in the tonoplast (Eom et al., 2015; Jung et al., 2015; Julius et al., 2017). BvTMT2.1, the sugar beet tonoplast monosaccharide transporter, plays a role as a specific transporter for sucrose uptake in the tonoplast; and therefore, it was included in the TST groups (Hedrich et al., 2015; Jung et al., 2015). The expression of these genes is strongly correlated with the accumulation of sucrose in plants (Julius et al., 2017). However, the expression of these genes was not specific to the IT-8 roots (Figure 5 and Supplementary Figure 7). These results supported our hypothesis that the high sucrose content in the roots of IT-8 could be transient.

### Sucrose Transport Pathway in Radish Roots

A positive *R* score between the gene *RSG17864* encoding CWINV and the sucrose content, and the distinct expression of the SUC1-encoding genes *RSG49762* and *RSG21948* and STP1-encoding gene *RSG00099*, among the sugar transporters, could provide information on the transport pathway of sucrose in radish roots (Figure 6B and Supplementary Figure 7). Sucrose degradation by CWINV in the apoplast and the cellular uptake by the hexose transporters constitute the apoplasmic pathway, which is essential for the apoplastic phloem unloading of sucrose by SUC1 transporters (Julius et al., 2017). In this study, the expression levels of two SUC1-encoding genes showed a positive *R* score when compared to that of the STP1-encoding gene (Supplementary Figure 7D). These results suggest that sucrose transport from leaves to roots is majorly mediated by the apoplasmic pathway in radish. However, the high sucrose content in the IT-8 roots cannot be explained by the apoplastic pathway. A positive *R* score between the sucrose content and the CWINV-encoding gene was achieved when IT-8 was excluded (Supplementary Figure 6). Sucrose transport can also occur via the symplastic pathway via plasmodesmata (Giaquinta, 1979; Fugate et al., 2019) and this pathway is considered the most important for the sink organs in plants (Verbančič et al., 2018). Therefore, the symplastic pathway could be partially involved in the high sucrose content of IT-8 roots.

### Membrane-Associated Sucrose Synthases and Cellulose Synthesis in Radish Roots

The high expression levels of SUSY1-encoding genes in JP-61 roots suggest that SUSY can act independently in the insoluble regions of cells (Figures 5B,D). SUSY exists in both soluble and insoluble forms, and the latter is mainly membrane-bound (Coleman et al., 2009). SUSY1 protein was detected in the insoluble material of Arabidopsis (Barratt et al., 2009). SUSY antibodies cross-react in membrane fractions of *Zea mays*, *Oriza sativa*, and *Nicotiana tabacum* (Persia et al., 2008). The resulting UDP-G by SUSY is a precursor for the synthesis of callose and cellulose; and therefore, SUSY is directly responsible for cell wall synthesis in the membrane pool. There is an association between SUSY and the membranes during the cell wall synthesis in plants (Bieniawska et al., 2007; Endler and Persson, 2011; Rende et al., 2017; Verbančič et al., 2018). SUSY is active in the cell wall of tobacco pollen tubes and is involved in the synthesis of cellulose and callose (Persia et al., 2008). More than half of the total SUSY is tightly associated with the plasma membrane in cotton fibers, and these play a role in the direct transfer of carbon to CSC or CALS (Amor et al., 1995; Stein and Granot, 2019). The overexpression of SUSY enhanced cellulose synthesis in cotton, and in a spontaneously recessive shrunken kernel maize mutant (shrunken1); the lack of SUSY activity decreased the cell wall thickness during endosperm development in transgenic poplar plants (Endler and Persson, 2011; Rende et al., 2017). The SUSY overexpressing transgenic hybrid poplar (*Populus alba* × *grandidentata*) exhibited an increase in the cellulose content from 2 to 6% (Coleman et al., 2009). Membrane-associated SUSY plays a role in inducing carbon flow during cell wall synthesis (Baroja-Fernández et al., 2012). In this study, high expression levels of CESA-encoding genes were identified in seven radish roots, which indicated that SUSY could act in the membrane pool and was closely related to cellulose synthesis.

### Alternative Pathway for Cellulose Synthesis in Radish Roots

SUSY plays an important role in cellulose synthesis; however, it is still unclear whether SUSY is essential for cellulose synthesis. Hybrid aspen (*Populus tremula* × *tremuloides*), which showed only 4% SUSY activity compared to that in the wild type, has normal cellulose biosynthesis (Rende et al., 2017). In Arabidopsis, the SUSY1/SUSY2/SUSY3/SUSY4 quadruple mutant, excluding SUSY5 and SUSY6, which are specifically related to callose synthesis in phloem sieve elements, are morphologically normal and there is no difference in cellulose content, compared to that in the wild type (Bieniawska et al., 2007; Barratt et al., 2009). The remained SUSY activity in this quadruple mutant is controversial (Baroja-Fernández et al., 2012); however, Barratt et al., 2009 suggested that SUSY is not essential for cellulose synthesis, and an alternative pathway could completely supply UDP-G for cellulose synthesis, therefore, replacing the deficiency in SUSY activity (Barratt et al., 2009). An alternative pathway for UDP-G production in sucrose metabolism is achieved through CINV and UGPase (Verbančič et al., 2018). Unlike the SUSY quadruple mutant, double mutants of CINV1 and CINV2 show significant inhibition of root and shoot growth, starch loss in the root cap, and changes in sugar levels, in Arabidopsis (Barratt et al., 2009). Abnormal cellulose biosynthesis and a decrease in UDP-G production were identified in this Arabidopsis mutant (Barnes and Anderson, 2018). In a CINV mutant of *Lotus japonicas*, the growth was seriously affected; inhibition of CINV in hybrid aspen reduced the level of UDP-G and reduced the crystalline cellulose content of woody tissue (Rende et al., 2017; Verbančič et al., 2018). UGPase is a key enzyme that produces UDP-G for carbohydrate metabolism via an alternative pathway (Kleczkowski et al., 2004). An Arabidopsis UGPase1/UGPase2 double mutant showed a growth defect and decreased levels of CESA transcripts (Endler and Persson, 2011). These results imply that CINV and UGPase are more important than SUSY in supplying UDP-G for cellulose synthesis in plants. However, our study showed a strong relationship between cellulose synthesis and the transcription of SUSY1. Genes encoding CINV and UGPase showed low expression compared to that of the two SUSY1-encoding genes, while CESA-encoding genes, involved in cellulose synthesis, were expressed at remarkably high levels, compared with that of callose and starch synthases (Supplementary Figure 5 and Figure 8). The *CINV* gene is highly expressed in 7-day-old radish roots, compared to that of the two *SUSY1* genes; however, the expression decreased in the 14-day-old radish roots. The two *SUSY1* genes were highly expressed, compared to that of the *CINV* gene in 14-day-old radish roots; and the strong expression of the two *SUSY1* genes was maintained during the development of radish roots (Mitsui et al., 2015). *CINV* seems to play an important role in the early stages of root development for UDP-G supply, and this role is likely to be replaced by *SUSY1* in radish roots. Sucrose catabolism by SUSY typically consumes less ATP than that by invertases, which is a reversible mechanism that allows feedback regulation. Therefore, this mechanism is advantageous in terms of energy conservation (Barratt et al., 2009).

### Sugar-Sensing in Radish Roots

When sucrose is degraded by SUSY, the resulting fructose causes an increase in the concentration of fructose in the cytoplasm. High fructose concentrations inhibit SUSY and FK activities in plants (Granot et al., 2013; Stein and Granot, 2018). Inhibition of these genes by high fructose concentration could play a role in diverting carbon into metabolic pathways other than cytoplasmic glycolysis (Stein and Granot, 2018); however, this phenomenon needs to be explained in terms of photosynthetic regulation through hexose sugar sensing. Sugars, including glucose and fructose, could be used as substrates for HK, and they inhibit the expression of genes involved in photosynthesis, such as Rubisco and chlorophyll A/B binding protein (Rolland et al., 2002; Granot et al., 2013). Tomato mutants that inhibit FK activity in petioles and stems accumulate less sugar in the stem, because of the reduced sugar transportation from the leaves (Stein et al., 2016). In this study, fructose content was negatively correlated with the expression levels of SUSY1, FK1, and PGI-encoding genes (Figure 6B). The high fructose concentration in the cytoplasm could have inhibited the photosynthetic activity of the source leaves, therefore limiting the sucrose transport to sink roots. Therefore, the expression of genes encoding SUSY1, FK1, and PGI was reduced in radish roots. Sugar sensing is also caused by glucose levels (Rolland et al., 2002; Eveland and Jackson, 2012; Granot et al., 2013). In this study, the glucose content showed a negative *R* score with the expression levels of the SUC1-encoding genes, which suggests that the high concentration of glucose in the cytoplasm limits the apoplastic phloem unloading of sucrose, by inhibiting the expression of SUC1 (Supplementary Figure 7C).

### Hexose Accumulation and Fructose Metabolism in Radish Roots

**FIGURE 9:**
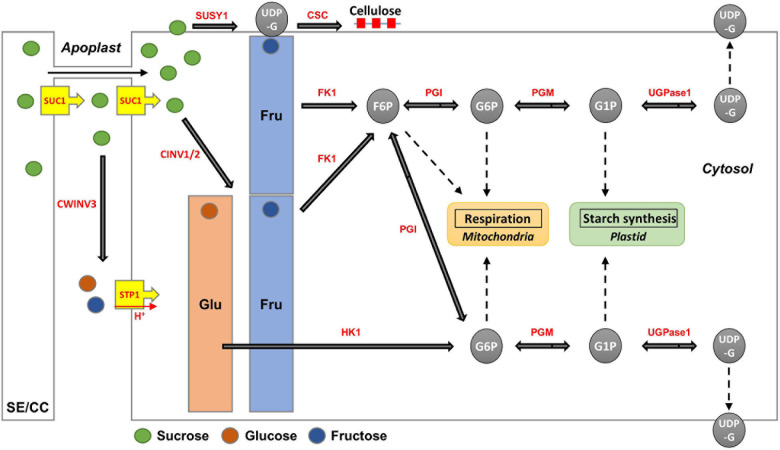
Putative schematic diagram of hexose accumulation and sucrose metabolism in radish roots. The major enzymes (red color) are encoded by the following genes: SUC1 encoding *RSG21948* and *RSG49762*, CWINV3 encoding *RSG17864*, STP1 encoding *RSG00099*, CINV encoding *RSG32129* and *RSG26046*, SUSY1 encoding *RSG05117* and *RSG17864*, FK1 encoding *RSG13222* and *RSG34089*, HK1 encoding *RSG19301* and *RSG43310*, PGI encoding *RSG20307* and *RSG14173*, PGM encoding *RSG15383* and *RSG26796*, and UGPase encoding *RSG31848*. CSC for primary cell wall formation is constructed by CESAs encoding *RSG00477*, *RSG06441*, *RSG00895*, *RSG02726*, *RSG00700*, *RSG09397*, *RSG17940*, and *RSG39758*.

The apoplasmic pathway can lead to a high hexose concentration in the cytoplasm (Julius et al., 2017). CINV is an essential enzyme for plant growth (Barratt et al., 2009; Barnes and Anderson, 2018). Bieniawska et al. (2007) hypothesized that all sucrose in the roots would be ultimately mobilized by invertases (Bieniawska et al., 2007). These suggest the importance of invertases for hexose accumulation and plant growth. In radish roots, the high hexose content is probably caused by invertases. Considering that hexose accumulation in the cytoplasm could be caused by invertases, the fructose produced by SUSY could be recognized as excess fructose. This hypothesis is supported by the fact that SUSY and FK work together (Stein and Granot, 2018), and our results corroborate this (Figures 7B,C). Additionally, positive *R* scores were found among the genes encoding FK and PGI (Figure 7D). Therefore, it is believed that most of the fructose produced by SUSY activity is phosphorylated in radish roots. In this process, the fructose produced by invertases could also be phosphorylated by FK1, which could explain why the fructose content in the cytoplasm is lower than the glucose content in most radish accessions (Figure 4). The invertases are less active than SUSY; however, the invertase pathway can supply carbon to all parts of sucrose metabolism through HK, PGM, and UGPase (Bieniawska et al., 2007). The less prominent activity of these genes is probably because SUSY is more efficient at carbon partitioning. In summary, we present a putative schematic diagram linking sugar content and sucrose metabolism in radish roots (Figure 9).

In conclusion, glucose is the dominant sugar in the radish roots. Fructose content correlated with the length, weight, and color phenotypes of roots. Two SUSY-encoding genes are highly expressed and are believed to be involved in cellulose synthesis. A high hexose content was possibly achieved via the apoplastic pathway. The significant *R* scores between the genes encoding SUSY and FK suggest that most of the fructose resulting from SUSY is phosphorylated. Sucrose was metabolized rather than stored, and cellulose was the main carbon sink in the radish roots. Additional molecular genetic studies are required to determine the correlation between phenotypic characteristic and gene expression. We believe that, these results might improve the overall understanding of sucrose metabolism in plants and provide information on important genes for developing new radish varieties.

## Data Availability Statement

The original contributions presented in the study are publicly available. This data can be found here: NCBI repository, accession number: PRJNA650223 (https://www.ncbi.nlm.nih.gov/bioproject/650223).

## Author Contributions

SML and S-JK contributed to the conception and design of the study. JSK provided the material for the study and contributed to the revision of the manuscript. M-SS collected materials and classified resources. SYW contributed to the review of the manuscript. J-NK wrote the first draft of the manuscript. All authors contributed to manuscript revision, read, and approved the submitted version.

## Conflict of Interest

The authors declare that the research was conducted in the absence of any commercial or financial relationships that could be construed as a potential conflict of interest.

## Publisher’s Note

All claims expressed in this article are solely those of the authors and do not necessarily represent those of their affiliated organizations, or those of the publisher, the editors and the reviewers. Any product that may be evaluated in this article, or claim that may be made by its manufacturer, is not guaranteed or endorsed by the publisher.

## Footnotes

1
https://ccb.jhu.edu/software/hisat2/index.shtml

2
https://pypi.org/project/HTSeq
