# Supplementary material for: Analysis of Phenotypic Characteristics and Sucrose Metabolism in the Roots of Raphanus sativus L
Source: Front Plant Sci. 2021 Oct 21;12:716782. doi: 10.3389/fpls.2021.716782 (PMC8566945; doi:10.3389/fpls.2021.716782)
Supplement: Supplementary file 2 [file Data_Sheet_2.docx]

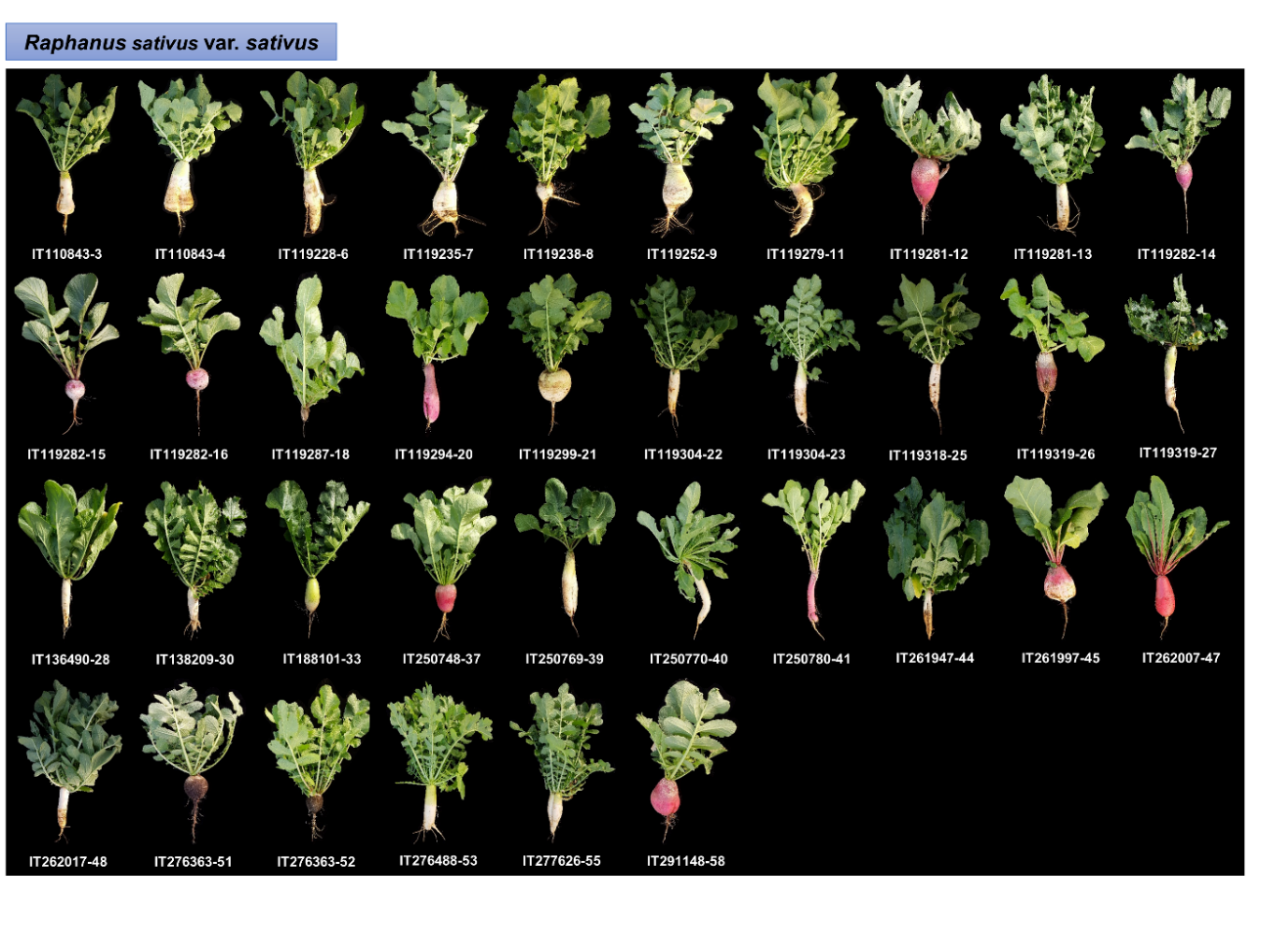

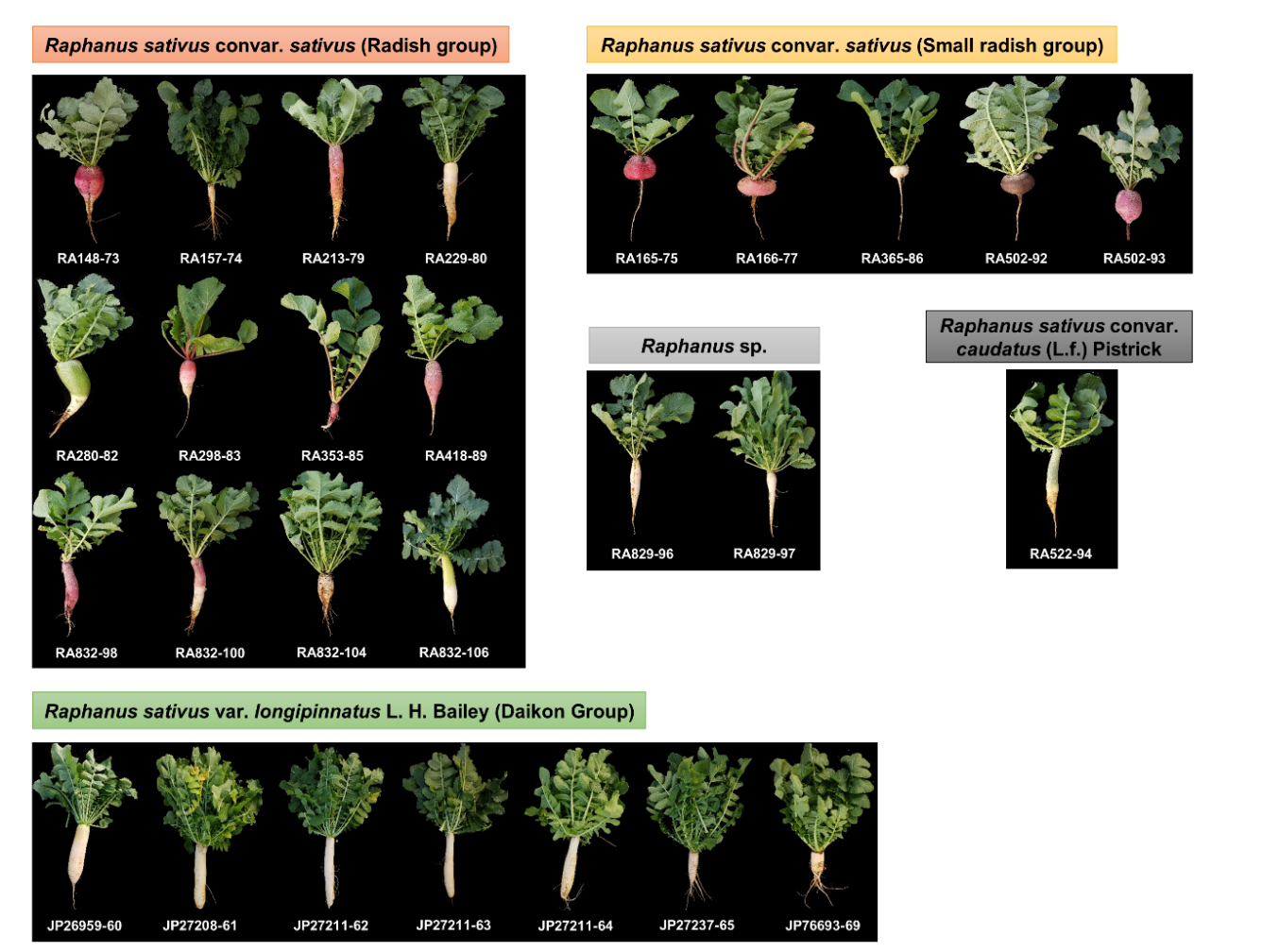


**Supplementary Figure 1.** Phenotypic images of the sixty three radish accessions.


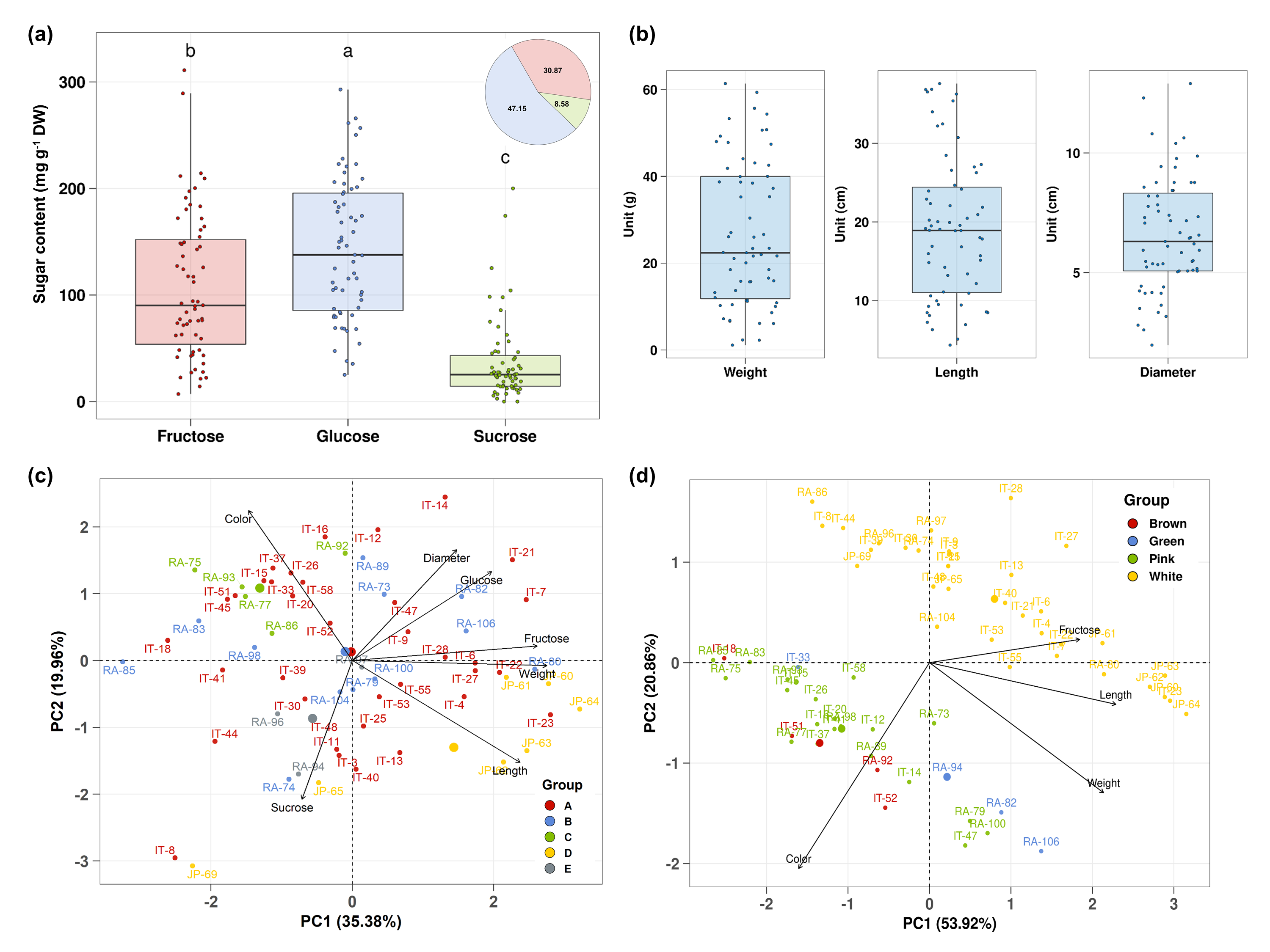


**Supplementary Figure 2.** Correlation analysis between the sugar content and the morphological characteristics of the root. Data visualization performed using R program. (a) Box plot analysis of the sugar content. Anova and Tukey Honest Significance Difference (HSD) test was performed to reveal differences among the sugar content. Other letters in the plot indicate significant differences at the *p* < 0.05 level. (b) Box plot analysis of morphological characteristics. (c) PCA plot based on five radish groups. A: *Raphanus sativus* var. *sativus*; B: *Raphanus sativus* convar. *sativus* (Radish group); C: *Raphanus sativus* convar. *sativus* (Small radish group); D: *Raphanus sativus* var. *longipinnatus* L. H. Bailey (Daikon Group); E: *Raphanus* sp. with *Raphanus sativus* convar. *caudatus* (L.f.) Pistrick. (d) PCA plot based on root color.


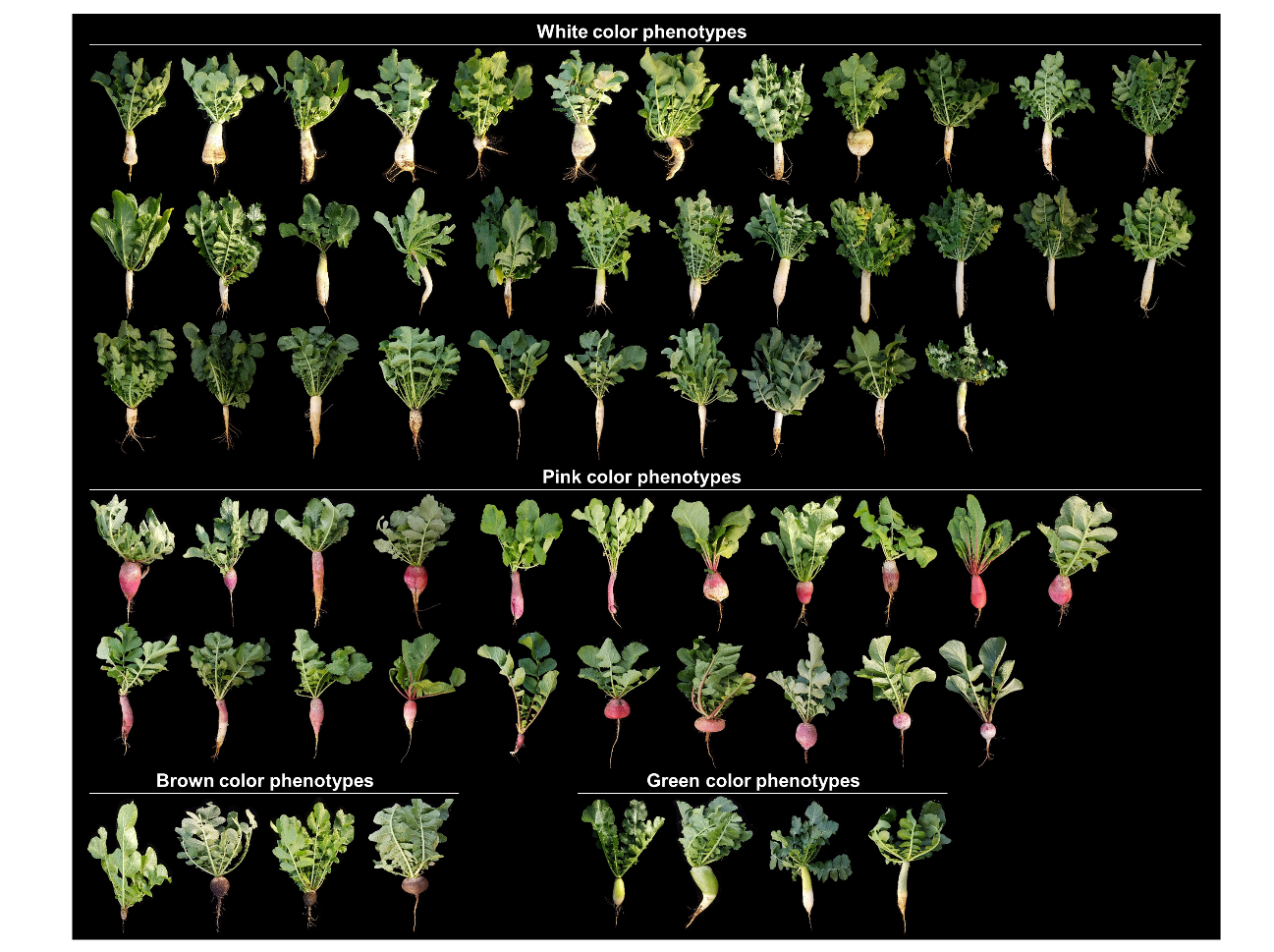


**Supplementary Figure 3.** Phenotypic images with grouping based on color in the sixty three radish accessions.


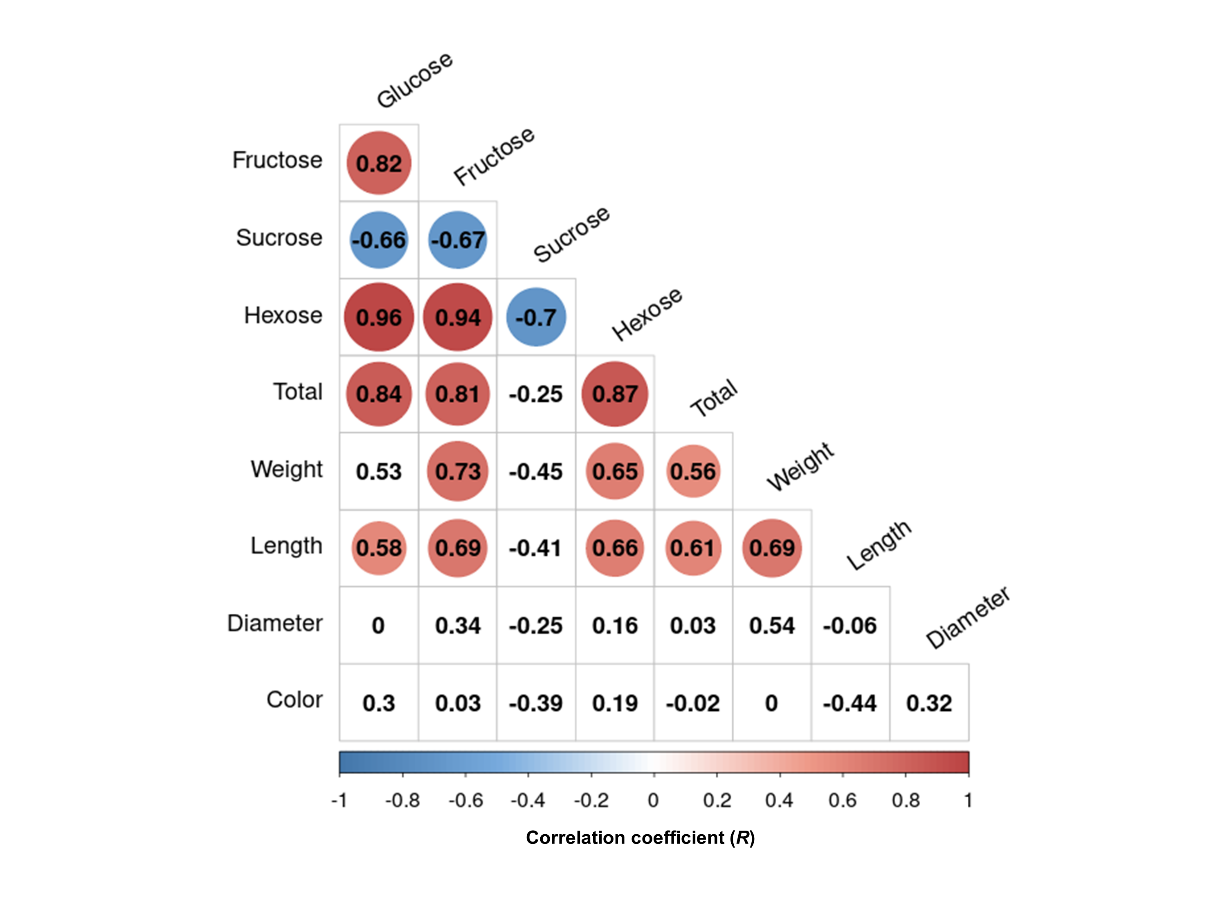


**Supplementary Figure 4.** Analysis of Pearson’s correlation coefficient (*R*) among the sugar content and root phenotypes in the seven radish accessions. Data visualization performed using R program. The *R* scores with *p* < 0.01 are indicated using color. Red and blue circles represent positive and negative *R* scores, respectively.


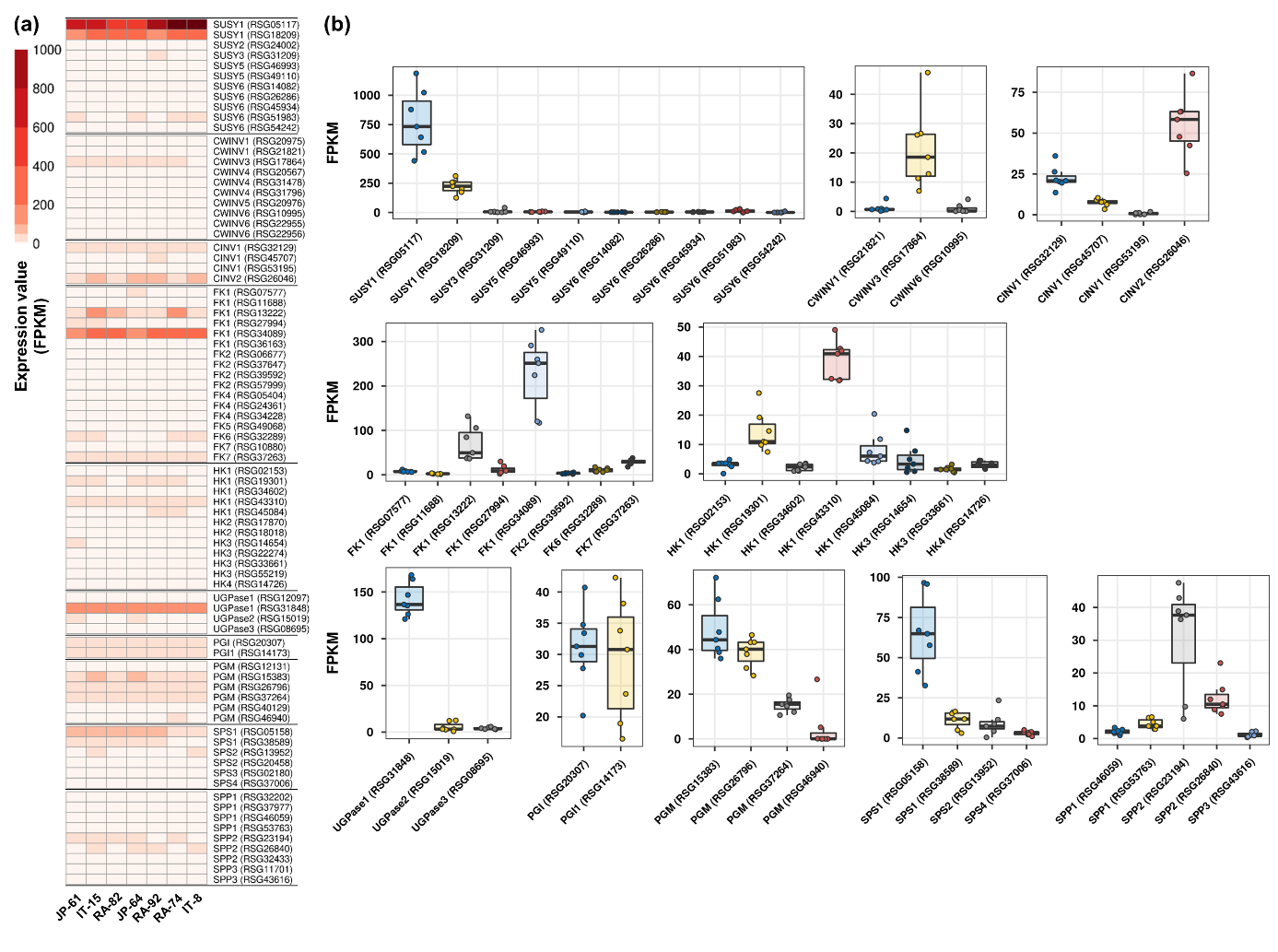


**Supplementary Figure 5.** Expression analysis of genes involved in sucrose metabolism. Data visualization performed using R program. (a) Analysis of heat map using FPKM. (b) Box plot analysis of the paralogue genes involved in sucrose metabolism.


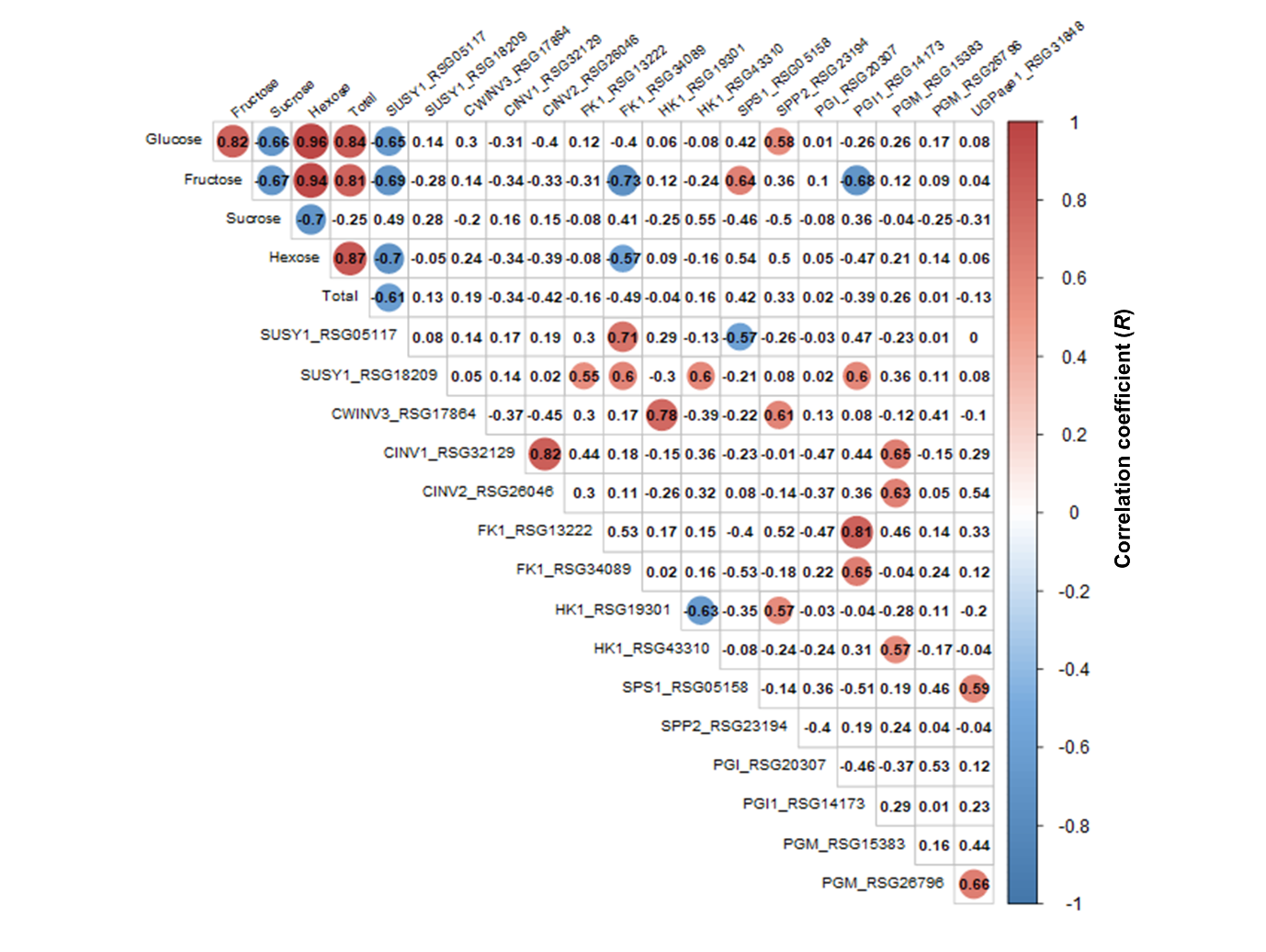


**Supplementary Figure 6.** Analysis of Pearson’s correlation coefficient (*R*) among the sugar content and the genes involved in sucrose metabolism in the seven radish accessions. Data visualization performed using R program. The *R* scores with *p* < 0.01 are indicated using color. Red and blue circles represent positive and negative *R* scores, respectively.


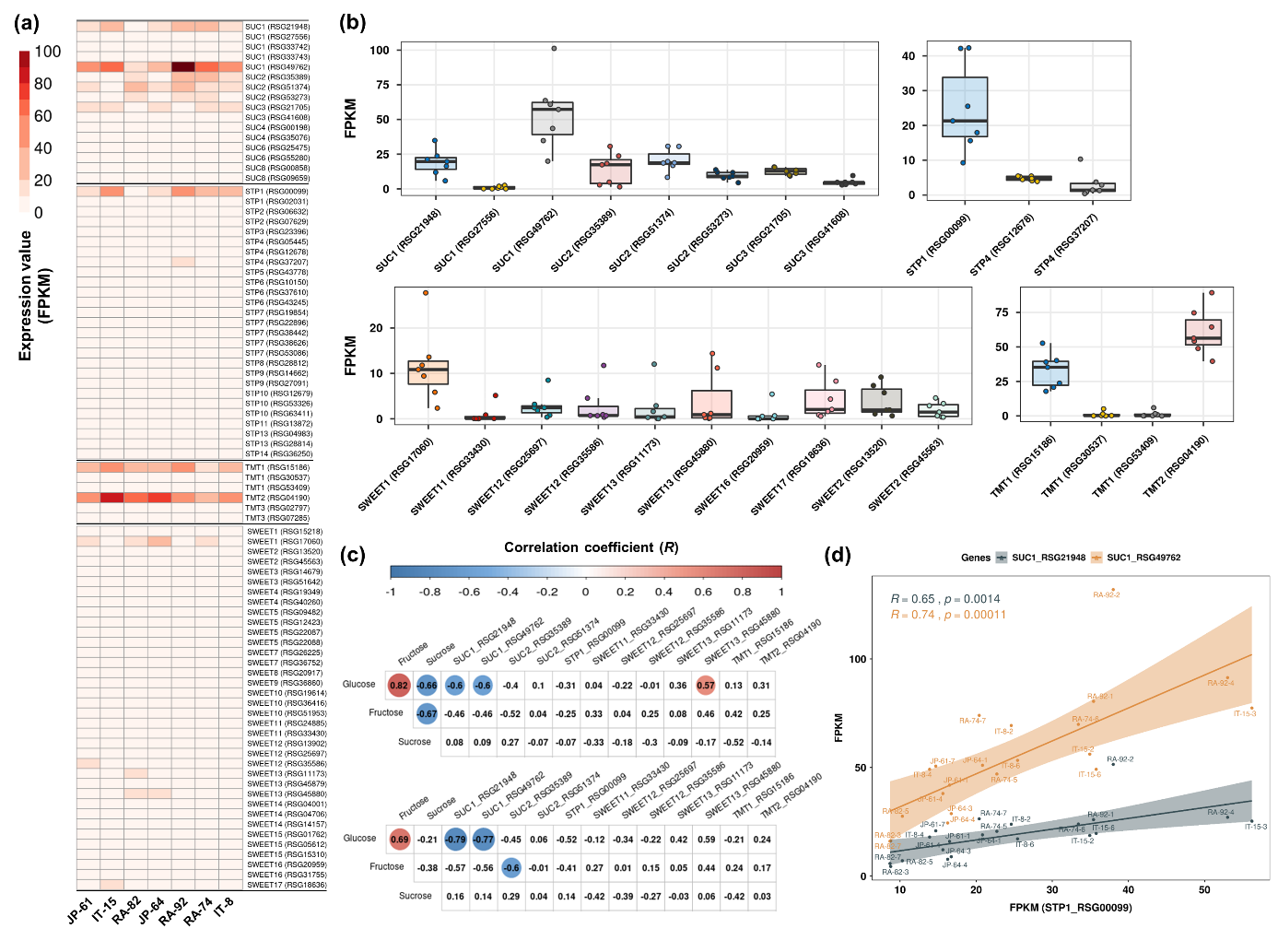


**Supplementary Figure 7.** Expression analysis of genes encoding the sugar transporter and Pearson’s correlation coefficient (*R*) with the sugar content. Data visualization performed using R program. (a) Analysis of heat map using FPKM. (b) Box plot analysis of the paralogue genes involved in sucrose metabolism. (c) Heat map of *R* score. The *R* scores with *p* < 0.01 are indicated using color. The below figure was excluding the IT-8 accession. (d) Correlation between the expression of the two SUC1-encoding genes and the STP1-encoding gene.
